# Multimodal landscape of atherosclerotic plaques: A spatial omics approach with mass spectrometry imaging

**DOI:** 10.1016/j.aca.2025.344723

**Published:** 2025-09-28

**Authors:** Robin Joshi, Soon Yew Tang, Ujjalkumar Subhash Das, Daniel J. Boehmler, Antonijo Mrčela, Ronan Lordan, E. James Petersson, Aalim M. Weljie, Garret A. FitzGerald

**Affiliations:** a Institute for Translational Medicine and Therapeutics, Perelman School of Medicine, University of Pennsylvania, Philadelphia, PA, USA; b Department of Systems Pharmacology and Translational Therapeutics, University of Pennsylvania, Philadelphia, PA, USA; c Department of Chemistry, School of Arts and Sciences, University of Pennsylvania, Philadelphia, PA, USA; d Department of Medicine, School of Medicine, University of Pennsylvania, Philadelphia, PA, USA

**Keywords:** Atherosclerosis, Mass spectrometry imaging (MSI), spatial omics, Lipids, Proteins, Artificial intelligence

## Abstract

Atherosclerotic plaques are complex and heterogeneous structures, originating as fatty streaks in the vasculature and formed by the accumulation of lipids and foam cells. Over time, these lesions progress as inflammation, smooth muscle cell proliferation and phenotypic switching, and extracellular matrix deposition contribute to plaque growth, culminating in their fracture, reactive thrombogenesis, and a cardiovascular event such as myocardial infarction and stroke. Traditional bulk mass spectrometry (MS) analysis has yielded critical insights into the molecular mechanisms of plaque formation and disease progression, but it is unable to determine the spatial heterogeneity and microenvironmental complexity within the lesion. Recent advances in mass spectrometry imaging (MSI) based omics, including spatial lipidomics, proteomics, and metabolomics, have enabled unprecedented visualization of molecular distribution in atherosclerotic plaques at cellular resolution. These techniques promise to elucidate the distinct cellular crosstalk, lesion vulnerability, and sex-specific disease mechanisms that contribute to plaque development and rupture. This review examines the recent advances in MS-based spatial omics and their application to atherosclerotic plaques in both experimental models and human samples. We highlight recent findings, explore their implications for precision medicine and translational research, and discuss current challenges in sample preparation and data integration. Despite challenges, we suggest approaches for integration of MS-based spatial omics using artificial intelligence (AI) to enhance data integration, interpretation, and translational applications in atherosclerosis research. These advances promise to broaden our understanding of atherosclerosis and identify novel therapeutic targets to limit the burden of cardiovascular disease.

## GRAPHICAL ABSTRACT

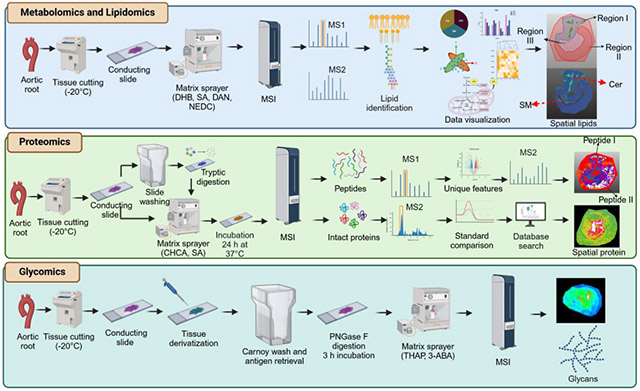

## 1. Introduction

**Fig. 1.**
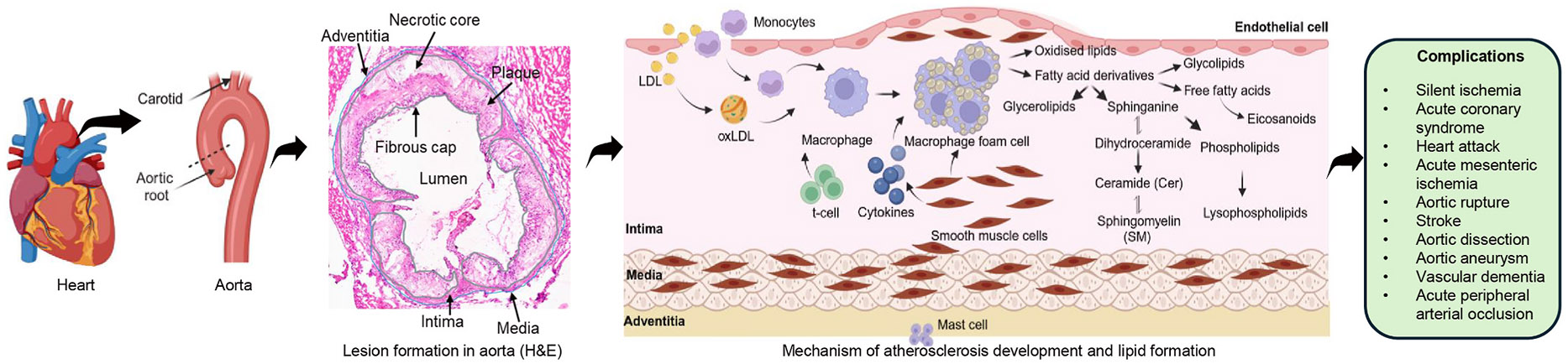
Schematic overview of initiation and progression of atherosclerosis in aorta.

Despite therapeutic advances, cardiovascular diseases (CVD) remain the leading cause of death in the United States [1]. At the root of CVD is the indolent progression of atherosclerosis, which is a chronic inflammatory disease of the vasculature characterized by infiltration of immune cells and lipids into the intima of medium to large blood vessels (Fig. 1). Over time, this process can lead to the formation of lesions that may progressively occlude blood vessels or rupture, activationg thrombogenesis and leading to myocardial infarction or stroke [2].

Single-cell RNA sequencing (scRNA-seq) and spatial transcriptomic technologies have unveiled the heterogeneity of cell types within atherosclerotic plaques in rodents and humans [3-7]. The comparative molecular heterogeneity of stable versus vulnerable plaques is revealed through spatially resolved expression of lipids, proteins, and metabolites [6-8]. This pathological process initiates and preferentially develops at sites within the vasculature subject to disturbed blood flow [9]. Studies in rodents have shown that vascular remodeling in response to disturbed blood flow accelerates atherosclerosis [10]. Unstable atherosclerotic plaques in these regions are enriched with oxidized lipids and infiltrated by multiple cell types, including inflammatory immune cells and fibroblasts [11-13]. Vascular smooth muscle cells (VSMCs) undergo phenotypic changes in response to inflammatory stimuli, transitioning from a contractile to a synthetic phenotype. This shift promotes extracellular matrix production, which influences plaque stability [14]. Endothelial cells (ECs) also become activated and, like other cells in the plaque, can synthesize and release oxylipins, including prostanoids and leukotrienes, which play essential roles in maintaining homeostasis, regulating immune responses, and modulating inflammation.

This diverse family of lipid mediators can exert both pro-and anti-inflammatory effects. Arachidonic acid (AA) is released from membrane phospholipids by the action of phospholipase A_2_ (PLA_2_) enzymes in response to diverse stimuli and serves as a substrate for cyclooxygenase-1 or -2 (COX-1 or COX-2) to form intermediate substrates (PGG_2_ and PGH_2_) from which are formed, prostanoids. Other enzymes that metabolize AA include lipoxygenases and cytochrome P450 [15,16]. Linoleic acid (LA) derived oxylipins, such as hydroxyoctadecadienoic acids (HODEs) and oxo-octadecadienoic acids (oxoODEs), also reflect and drive oxidative stress and vascular inflammation [17-19].

Given the coexistence of stable and vulnerable plaques within a microenvironment infiltrated by diverse cell types, mapping the spatial molecular profiles including the lipidome, proteome, metabolome, and transcriptome of the various cell types within these plaques could unveil new therapeutic targets to delay the progression of atherosclerosis. Since the inception of mass spectrometry imaging (MSI) in 1967 [20], the field of MSI has quickly grown with over 3500 manuscripts in PubMed documenting applications in various tissues, including atherosclerotic plaques [21]. Numerous reviews have focused on the emerging analytical advances of MSI in tissues such as the kidney, brain, and liver [22-25]. Here we focus on the integration of multiomics data with spatial cellular biology in preclinical models of atherosclerosis and in human tissue obtained at endarterectomy or postmortem.

First, we discuss advances in MSI relevant to high-spatial-resolution, localization of lipids, proteins and glycans in atherosclerosis. Secondly, we consider the application of these approaches to analysis of vascular tissues. Thirdly, as an example, we highlight the utility of MSI to analyze low abundant oxylipins in atherosclerosis. Finally, we offer some perspectives on artificial intelligence (AI) based data integration.

## 2. Advanced mass spectrometry imaging technologies in atherosclerosis

### 2.1. MALDI-MSI for spatial lipidomics and metabolomics

Matrix-assisted laser desorption/ionization mass spectrometry imaging (MALDI-MSI) has emerged as the most commonly used tool for exploring spatial lipidomics in atherosclerosis research. MALDI-MSI enables the direct analysis of lipid distributions in tissue sections without extraction. In MALDI-MSI, the tissue section is coated with a matrix such as 2,5-dihydroxybenzoic acid (DHB), α-cyano-4-hydroxycinnamic acid (CHCA), or 9-aminoacridine (9-AA). The matrix absorbs energy from a focused laser, leading to the desorption and ionization of analytes. The resulting charged analytes (positive or negative) are transferred to different types of analyzer such as quadrupole mass analyzers, ion trap analyzers, time of flight (TOF), and separated based on mass-to-charge (*m/z*) ratio [26]. After separation, ions are analyzed by a mass spectrometer to determine the time required for the ions to travel the length of the flight tube and generate the mass spectra at each pixel on the tissue section. By combining the spectra with spatial coordinates, a detailed map of metabolite, protein and lipid distributions across the tissue is generated [27].

Applications of MALDI-MSI in atherosclerosis research have used sophisticated approaches to map lipid metabolites with high spatial precision. For example, Cao et al. utilized both 2D and 3D MALDI-MSI at high spatial resolution (15 × 15 μm^2^ and 20 × 20 μm^2^) to characterize atheroma-specific lipids in mouse models of atherosclerosis [28]. They identified 11 lipid species that were significantly elevated in plaques, including one sphingomyelin (SM 34:0; 2), three lysophosphatidic acids (LPA 16:0, 18:1, 20:1), four lysophosphatidylcholines (LPC 16:0, 18:0, 18:1, 18:2), two lysophosphatidylethanolamines (LPE O-18:1, 18:0), and one lysophosphatidylinositol (LPI 18:0). Notably, through 3D volumetric evaluation, they demonstrated that LPI 18:0 was specifically localized to the necrotic core regions, while the other lysolipids distributed elsewhere in the plaque.

Moerman et al. extended this approach to human atherosclerotic tissue, using MALDI-MSI to visualize approximately 200 different lipid signals (originating from >90 uniquely assigned species) across 106 tissue sections from 12 carotid plaques [29]. Their experimental workflow combined MALDI-MSI with histological staining and multivariate statistical analysis to correlate specific lipid patterns with histological features of plaque vulnerability. They detected multiple lipid classes including cholesterol, cholesteryl esters (CEs), lysophosphatidylcholines, phosphatidylcholines, sphingomyelins, diacylglycerols (DGs), and triacylglycerols (TGs). Their analysis revealed that sphingomyelins and oxidized cholesteryl ester species, particularly 7-ketocholesterol, were significantly elevated in necrotic intima. Interestingly, they found that diacylglycerols and triacylglycerols were spatially correlated with areas containing fibrin, suggesting a potential mechanistic link to thrombus formation.

Both studies employed sophisticated data processing techniques, including spatial cross-correlation analysis, unsupervised clustering algorithms (e.g., non-negative matrix factorization), and histology-directed multivariate analysis (orthogonal projections to latent structures discriminant analysis) to extract biologically meaningful patterns from the complex MALDI-MSI datasets. These methodological approaches have significantly enhanced our ability to understand the spatial organization of lipid metabolites in atherosclerotic plaques and their potential roles in disease progression and complications.

Some key advantages of MALDI-MSI include its well-established use in biomedical research, faster data acquisition compared to some other MSI techniques, and high spatial resolution, with pixel sizes of less than 5 × 5 μm in some cases [30,31]. However, there are also some challenges and limitations. The choice of matrix is critical and can impact both spatial resolution and sensitivity. Different matrices also affect ionization efficiency and selectivity for different lipid classes, potentially introducing bias towards certain lipid populations [32]. Additionally, matrix application itself introduces an extra sample preparation step and potential source of variability. Furthermore, the laser used in MALDI tends to be relatively aggressive compared to other ionization techniques. If the laser intensity is too high, it can cause tissue destruction that may interfere with subsequent analyses of the same section [33]. MALDI-MSI is often used as a first-pass imaging technique, followed by other spatially-resolved analyses like histological staining, immunohistochemistry, or spatial transcriptomics. Removing the MALDI matrix to enable these downstream assays requires an additional step that risks damaging the tissue.

### 2.2. DESI-MSI for in situ lipidomics and metabolomics

Desorption electrospray ionization mass spectrometry imaging (DESI-MSI) is an emerging ambient ionization technique for spatial lipidomics and metabolomics. In contrast to MALDI, DESI-MSI allows direct analysis of tissue sections without the need for matrix application or extensive sample preparation (Table 1). In DESI-MSI, a focused spray of electrically charged solvent droplets is directed onto the tissue surface. These droplets interact with and extract analytes from the tissue, generating secondary microdroplets containing the desorbed ions. The ions are then transferred to the mass spectrometer inlet for analysis, producing a mass spectrum at each sampled location on the tissue.

DESI-MSI has been used to map metabolite distributions in atherosclerotic lesions and identify disease-associated spatial patterns. Slijkhuis et al. applied DESI-MSI to human carotid atherosclerotic plaques and correlated the spatial localizations of lipids with histologically-defined plaque regions [34]. They found that specific sphingomyelin (SM) and ceramide (Cer) species co-localized with calcification, phospholipids and free fatty acids were associated with inflammation, and triacylglycerols and phosphatidylinositols were enriched in fibrin-rich regions due to the influence of platelets in the lesions.

In a direct comparison of MALDI and DESI techniques for lipid imaging in atherosclerotic plaques, Slijkhuis et al. [35] found that DESI-MSI exhibited higher overall sensitivity for most lipid classes than MALDI-MSI and provided sharper images with improved feature definition, likely due to matrix induced delocalization [36]. Specifically, DESI showed greater peak intensities for cholesteryl ester and triacylglyceride species, likely due to reduced fragmentation in its softer ionization process. Quantitative assessment using spatial autocorrelation analysis confirmed that DESI images had 1.5–2 times better feature definition than MALDI images.

However, MALDI-MSI demonstrated superior capabilities for detecting certain lipid species, particularly ceramides, hexosylceramides, and lactosylceramides. This advantage was attributed to MALDI’s efficient generation of dehydrated ions ([M + H–H_2_O]^+^) for ceramide species, while DESI predominantly detected them as sodiated adducts ([M+Na]^+^). This finding underscores how the different ionization mechanisms can provide complementary information about the lipidome of atherosclerotic plaques.

**Fig. 2.**
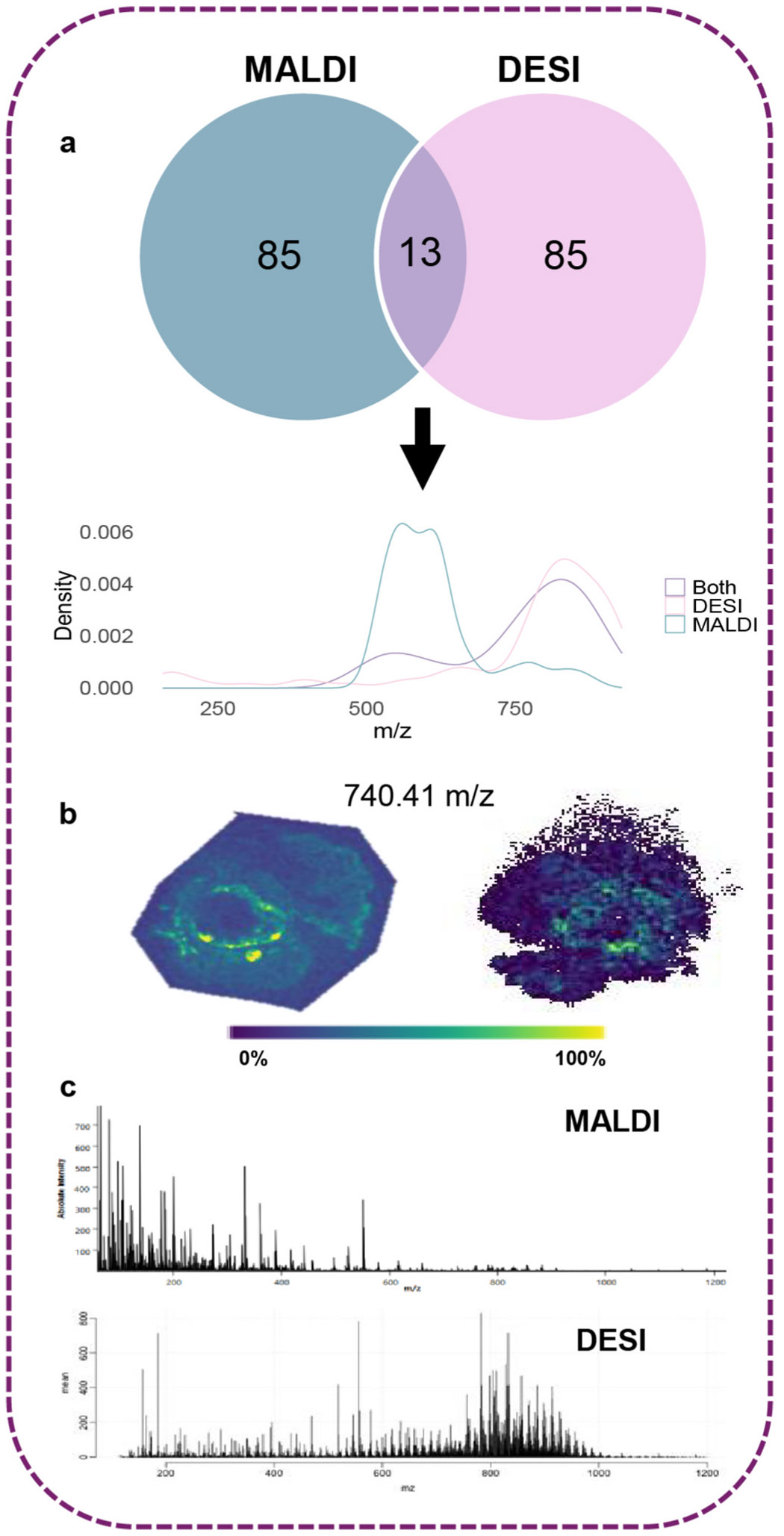
(a) Venn diagram comparing the top 98 features in the MALDI MSI spectrum and the top 98 features in the DESI MSI spectrum, along with a density plot of these features to demonstrate their unique mass distributions. **(b)** Visualization of a single ion localized to the aortic root of a diseased male mouse heart using both MALDI (left) and DESI (right). **(c)** A comparison of mass spectra obtained from diseased male mouse heart sections using MALDI and DESI.

For example, in atherosclerotic plaques of the aortic root of a male mouse, we compared the 98 most abundant features detected in MALDI-MSI data and the 98 most abundant features in DESI-MSI data. Of these, MALDI and DESI each showed 85 distinct features, while sharing 13 features (Fig. 2a and b). By observing their respective mass spectra and the feature density of the highest abundance ions, it is clear that the two methods detect complementary mass ranges; MALDI data has a greater number of features in the range *m/z* 500–700, while DESI has a higher feature density in the higher ranges (*m/z* 700–1000), where most phospholipids can be detected (Fig. 2a and c).

Compared to MALDI-MSI, DESI-MSI offers some complementary advantages, including gentler ionization that causes less tissue damage, preserving tissue integrity for follow-up analyses; a simpler workflow with minimal sample preparation, reducing potential user variability; and greater amenability to combination with other spatial assays on the same tissue section. However, DESI-MSI also has some limitations. DESI-MSI generally has longer acquisition times to scan a tissue section and a somewhat lower spatial resolution compared to MALDI-MSI. However, recent advances have significantly narrowed this gap. A multimodal workflow has been successfully integrated combining pneumatically assisted nanospray desorption electrospray ionization (PA nano-DESI) MSI and Raman spectroscopy on the same rat kidney tissues [37]. Another multimodel method has been established that integrates stimulated Raman scattering (SRS) microscopy, desorption electrospray ionization (DESI) and MeV ion beam analysis (IBA) on the same fresh frozen porcine skin tissue section [38]. The composition of the spray solvent also affects extraction and ionization of different metabolite classes, so solvent selection can bias the resulting molecular images, a parallel to matrix composition effects in MALDI-MSI acquisition.

### 2.3. SIMS-MSI for high resolution molecular mapping

Secondary ion mass spectrometry imaging (SIMS-MSI) is a spatial resolution technique that uses a focused primary ion beam to ionize and desorb molecules from the sample surface. The resulting secondary ions are then analyzed by mass spectrometry to generate spatially-resolved molecular maps [22]. SIMS-MSI can achieve submicron spatial resolution, enabling detailed localization of lipids and metabolites at the cellular and subcellular scale. In atherosclerosis research, SIMS-MSI has been employed to visualize lipid distributions with high precision in both human and mouse plaques. Lehti et al. used TOF-SIMS to analyze the spatial distribution of lipids in human coronary artery cryosections [39]. They discovered that the ratio of cholesterol fragment ions (*m*/*z* 385: *m*/*z* 369) could differentiate unesterified cholesterol from cholesterol esters, demonstrating changes during atherogenesis and in different plaque regions. Their studies revealed that atheromas were characterized by accumulation of cholesterol esters with apolipoprotein B near the intima-media border, while in complicated lesions, unesterified cholesterol dominated in neovessel-containing areas enriched in glycophorin A. Notably, they found a spatial segregation of cholesterol and triacylglycerols, with triacylglycerols primarily located in areas surrounding neo-vessels and lacking either form of cholesterol.

A major advantage of SIMS-MSI is its superior spatial resolution compared to other MSI techniques, which allows for precise correlation of molecular signatures with tissue morphology at the microscopic level. However, SIMS does have some drawbacks for lipid and metabolite analysis. The high energy primary ion beam causes extensive fragmentation, so most of the detected secondary ions are fragments rather than intact molecular ions. This complicates metabolite identification due to a low molecular ion yield [40]. The ion beam destroys the sample surface during analysis, making it difficult to analyze the same tissue section again or use certain downstream assays [41]. SIMS also has somewhat lower chemical sensitivity compared to MALDI and DESI, especially for larger biomolecules, though advances like cluster ion sources have improved this [42].

Indeed, MSI technologies like MALDI, DESI, and SIMS are providing unprecedented insight into the spatial organization of lipids and metabolites within atherosclerotic plaques (Table 1). In addition to the above MSI techniques, a highly sensitive atmospheric pressure laser ablation carbon fiber ionization mass spectrometry imaging (AP-LACFI-MSI) method has been developed to enable spatial distribution analysis of secondary metabolites [43]. While each technique has particular strengths and limitations, they offer complementary perspectives for probing atherosclerosis biology across different spatial scales. Combining MSI with other imaging modalities and omics technologies promises to paint an ever more complete picture of the metabolic drivers of atherosclerotic disease.

## 3. Proteomic and glycomic imaging

### 3.1. Advances in spatial proteomics using MSI

Protein biomarkers have been investigated in tissue and plasma from patients with atherosclerosis using high throughput proteomic platforms for the evaluation of cardiovascular risk factors, therapeutic targets, and clinical applications [44-46]. In vascular tissues for example, a proteomic atlas was recently developed, based on tissue from 200 patients undergoing carotid endarterectomy [6]. Attention has now shifted towards getting precise and spatially resolved proteomic information from different regions of the same or sequential tissue sections. Automated MSI enables high-throughput mapping of over 2000 proteins from tissue sections with a spatial resolution of 100 μm [47,48]. Another study has shown how deep-learning-based imaging spatial proteomics (DISCO-MS) can be used to parse the heterogeneity of immune cell-enriched tissues from bone marrow niches of intact mice to aortic plaques from the human heart [49]. For example, myosin heavy chain (MYH8) was found to be upregulated (2.2-fold, *p* < 0.05) in atherosclerotic plaque, along with MYH10 and MYH11. These have now been put forward as potential biomarkers of atherosclerotic plaque formation [49,50]. TGF-β1 was the most prominent target protein related to fibrosis in plaque [51]. MALDI-MSI and immunohistochemistry (IHC) also revealed a significant increase in the expression of thymosin β4 (TMSB4X, *m/z* 4762), a protein located in the intima of atherosclerotic rabbit and human aortas compared to healthy tissues [21].

### 3.2. Advances in glycomics using MSI

Glycans are molecules that contain glycosidic bonds encompassing sugars (e.g. monosaccharides, oligosaccharides, polysaccharides) and their carbohydrate portions bound to proteins or lipids. Complex enzymatic pathways comprising glycosyltransferases and glycosidases, are responsible for the formation of glycans. Glycan signals are likely to reflect inflammation [52]. For example, high-mannose glycan excretion in urine has been suggested as a predictor of cardiovascular events in patients with type 2 diabetes. Sequential MSI of lipids, N-glycans, and tryptic peptides on a single formalin-fixed paraffin-embedded (FFPE) tissue section illustrates the enhanced molecular characterization achieved by the acquisition of multiple modalities on the same slide [53]. Enzymatic regulation of glycan-mediated posttranslational modifications has also been used to identify novel biomarkers and biological pathways that may be relevant for atherosclerosis and cardiovascular events [54]. GlycA has been associated with peripheral artery disease, extra-coronary calcifications in cardiac valves and the thoracic aorta, as well as subclinical femoral and carotid plaques [55]. The glycosylation profile of two independent cohorts showed that immunoglobulin G is cross-sectionally associated with subclinical atherosclerosis and cardiovascular risk [56]. MALDI-MSI is a fast and robust method for analyzing the spatial distribution of glycans in FFPE human tissue samples [57]. Elevated sialylated glycans have been reported in stenotic aortic valves [58]. Disease-driven modifications of N-glycan structures may disrupt cell-cell recognition, migration, and proliferation, leading to pathological changes in key signaling pathways, including TGFβ1, ERK, and EGFR [59,60]. Three sialylated N-glycans exhibited consistent elevation and distinct localization in stenotic aortic valves [61]. The heterogeneity amongst clinical samples presents a particular challenge to segregating molecular characteristics to distinct regions, implying functional differentiation. MSI protein and glycan ion signals may vary due to heterogeneity within the tissues or the donors. Proteins and glycans may have suppressed ionization by giving weak signals in highly complex regions. This can highlight unique regional expression patterns that would be obscured if tissues were treated as homogeneous. Differences in cellular composition, matrix properties, and metabolite concentrations within a tissue section can lead to analytical artifacts such as ion suppression, reduced sensitivity, and poor spatial segmentation. These factors contribute to signal variability, spatial concentration differences, and difficulty in resolving co-localized molecules, ultimately complicating data interpretation. Such challenges can be addressed through improved sample preparation strategies, advanced computational approaches, multimodal imaging, and data integration. Furthermore, advances in high-resolution techniques, such as tissue expansion mass spectrometry imaging (TEMI), enable deeper investigation of cellular heterogeneity [62]. Aside from heterogeneity within the samples, heterogeneity of the degree of disease progression is often apparent in clinical samples where sample acquisition is often opportunistic compared with staged sampling of atherogenesis during preclinical studies [63-65].

Different groups of researchers used diversified imaging approaches (Rapiflex, TIMS-TOF-MS, DESI-MS, SIMS, nano-DESI, and MALDI-FTICR), advanced software (Fleximaging, Cardinal, SCiLS, META-SPACE, JuliaMSI [66], MSI Eagle [67], mzmine, LipidXplorer) and workflows to identify and quantify the proteins in carotid and aortic arteries.

## 4. Challenges in imaging atherosclerosis across different tissues

### 4.1. Vascular heterogeneity and molecular fingerprinting

**Fig. 3.**
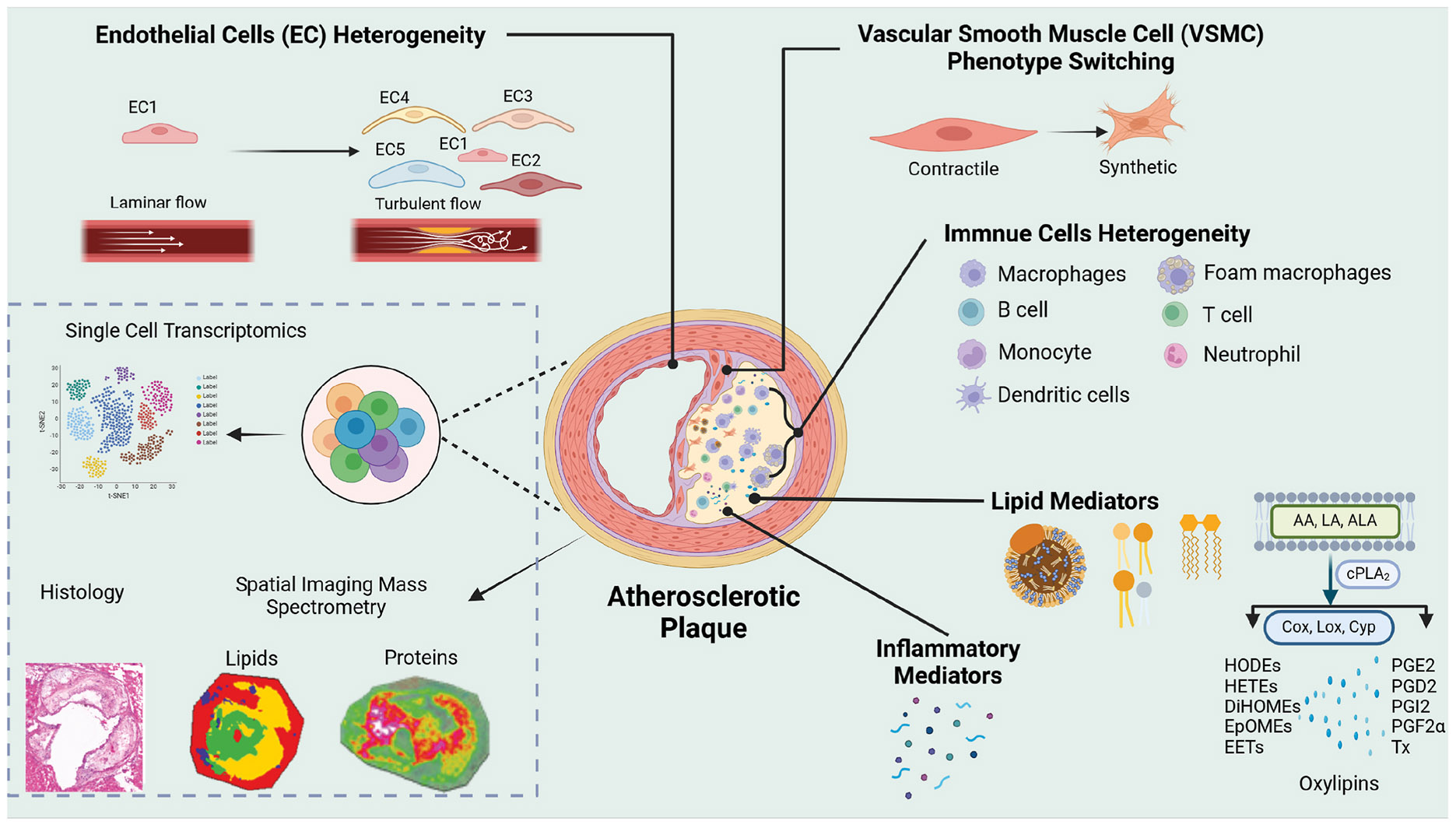
A schematic overview of cellular heterogeneity in atherosclerotic plaque and vascular wall.

Spatial MSI is a powerful tool for mapping the biochemical landscape of atherosclerosis, revealing plaque heterogeneity by visualizing lipids, metabolites, and proteins. However, atherosclerotic lesions contain diverse cell types that distinctly contribute to disease pathogenesis (Fig. 3).

Cao et al. identified lipid metabolites such as LPC (18:0) and LPA (18:1) in advanced plaques, suggesting their potential as biomarkers for cardiovascular risk [28]. This approach links lipid profiles with plaque characteristics, offering insights into lipid metabolism in atherosclerosis. Greco et al. demonstrated region-specific lipid variations in symptomatic carotid plaques, suggesting their impact on plaque stability [68]. The high spatial resolution of MSI is crucial for identifying vulnerable plaques prone to rupture. Seeley et al. correlated molecular data with histological features to identify metabolites associated with plaque vulnerability, offering a comprehensive perspective on atherosclerosis progression [8].

### 4.2. Challenges in analyzing fibrous vs lipid-rich atherosclerosis plaques

Fibrous plaques, characterized by a thick fibrous cap composed of VSMCs and extracellular matrix, provide structural stability [69,70]. In contrast, lipid-rich plaques contain large necrotic cores filled with lipids and macrophages, making them more prone to rupture [71,72]. Lipid-rich plaques often exhibit heightened inflammatory activity, with macrophage infiltration and pro-inflammatory cytokines weakening the fibrous cap and increasing the risk of rupture [73,74]. Though fibrous plaques are generally stable, they can also undergo pathological changes, such as VSMC apoptosis and extracellular matrix degradation, which may compromise their integrity [75]. This variability complicates interpretation of MSI data, as different plaque regions exhibit distinct lipid and metabolite profiles. Seeley et al. found that stable plaques are enriched with very long chain fatty acids [8], whereas unstable plaques had elevated oxidative stress and inflammation-related metabolites such as 5-hydroxyindoleacetic acid [29,68]. MSI lipid profiling within plaques reveals region-specific alterations correlated with stability [28,68]. For example, symptomatic plaques contain higher levels of sphingomyelins and cholesterol esters, markers of increased lipid uptake and inflammation [68]. The interaction between plaque composition, inflammation, systemic circulating lipids and hemodynamics further complicates analysis. Paradoxically, low shear stress regions tend to accumulate lipids and recruit inflammatory cells, promoting lipid-rich plaque formation [76].

### 4.3. An example of tissue preparation for MSI using aortic root from mouse

**Fig. 4.**
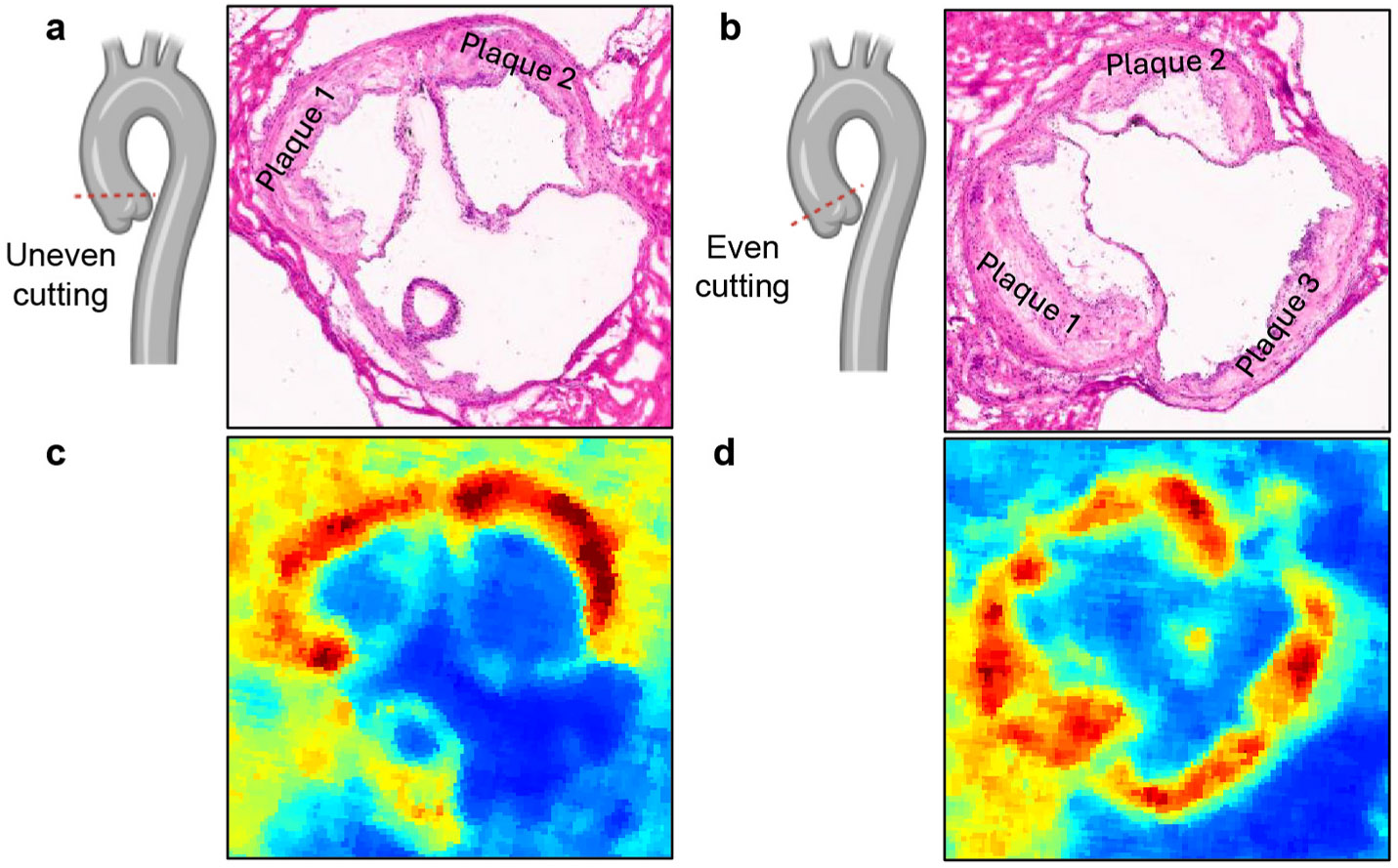
Uneven **(a, c)** and even **(b, d)** aortic root sections cutting using cryostat (−20 °C) followed by H&E staining and MALDI ion images, respectively.

Studying atheroscloretic plaques in the aortic root in mouse models of atherosclerosis illustrates some of the tissue-based challenges to MSI analysis. First, there are anatomical differences (i.e. the three leaflets of the aortic valve are often involved to different degrees) that must be addressed for lesion quantification and comparison with controls. Fig. 4a and c displays a section of the aortic root stained with hematoxylin and eosin (H&E), where one of the three leaflets was misaligned during sectioning, in contrast to a section where leaflets were properly aligned as shown in Fig. 4b and d.

Working with aortic roots from mice with advanced atherosclerosis presents another challenge due to the presence of two distinct compartments – the underlying vascular structures - ECs, VSMCs, and adventitia cells – and the superimposed lipid deposition, immune cells and fibroblasts. To mitigate the cracking of tissues during cryosectioning, advanced atherosclerotic plaques (for example, ≥9 and ≥ 12 months of Western diet (WD) in male and female low-density lipoprotein receiptor deficient mice on C57BL6/J background, respectively) should be sectioned at a temperature below −20 °C using embedding reagents compatible with these low sectioning temperatures.

**Fig. 5.**
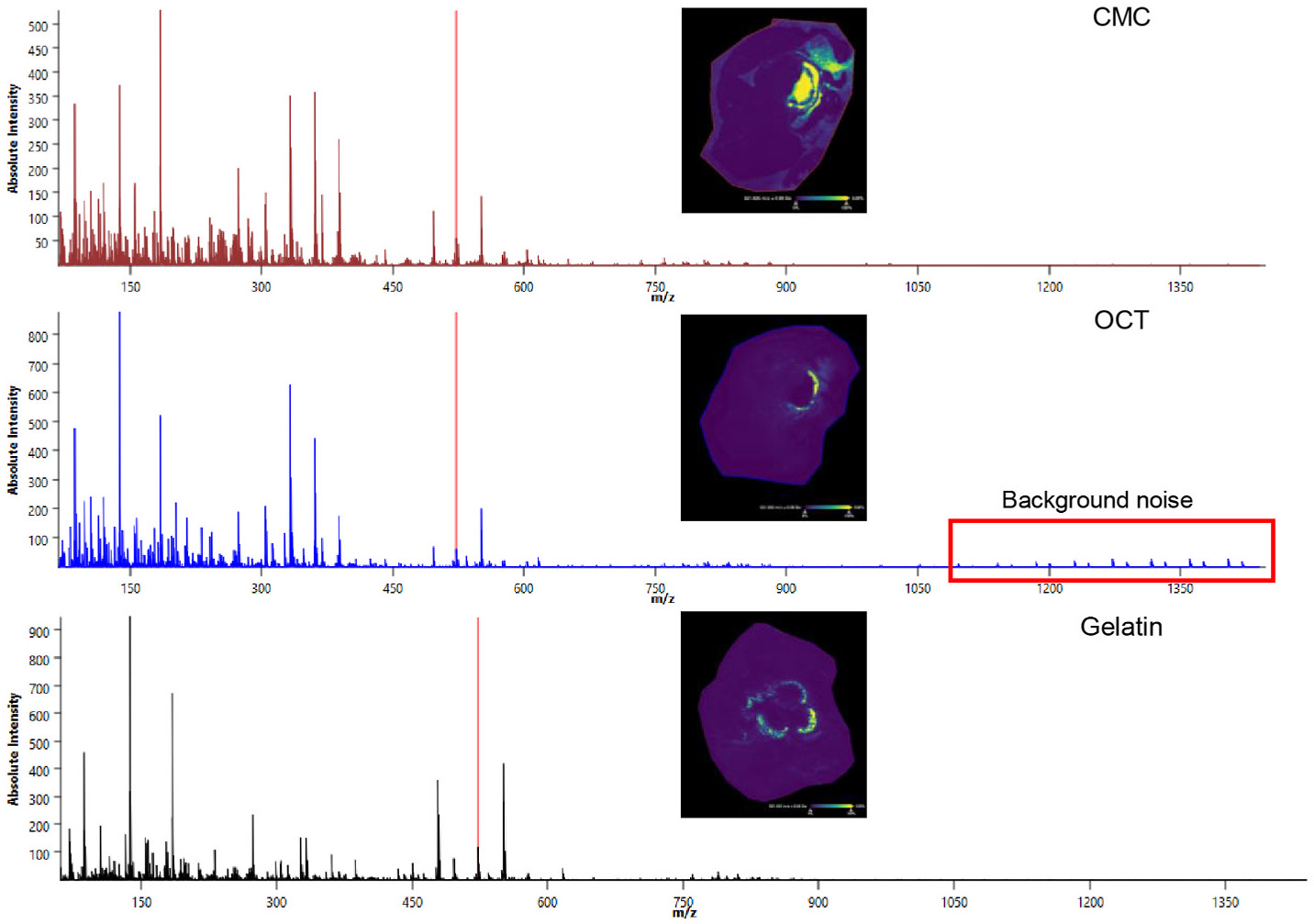
Comparative aortic root sections showing the spatial distribution and relative intensities in CMC, OCT, and gelatin embedding reagent using MSI. Background noise was noticed with OCT embedded tissue and is highlighted in red rectangle.

In this example, the mouse heart tissue (top 1/2) containing the aortic root was placed in a mold and embedded with fish gelatin (12.5 %). One of the most common embedding reagents, optimal cutting temperature (OCT) reagent, is not suitable for imaging using MALDI and DESI, as it produces background signals that interfere with the acquired data (Fig. 5). Carboxymethylcellulose (CMC) is an alternative embedding reagent for various tissues including aortic roots. To achieve high-quality tissue sections, a mixture of various embedding reagents can be used based on the characteristics of the tissue. For example, adipose tissue can be embedded in gelatin: CMC: H_2_O mixture (10:5:85 % w/v) and sectioned at −35 °C [77]. Formalin fixed tissue is typically not recommended for use in spatial lipidomic studies due to the potential loss of certain lipid species during the dehydration processes.

**Fig. 6.**
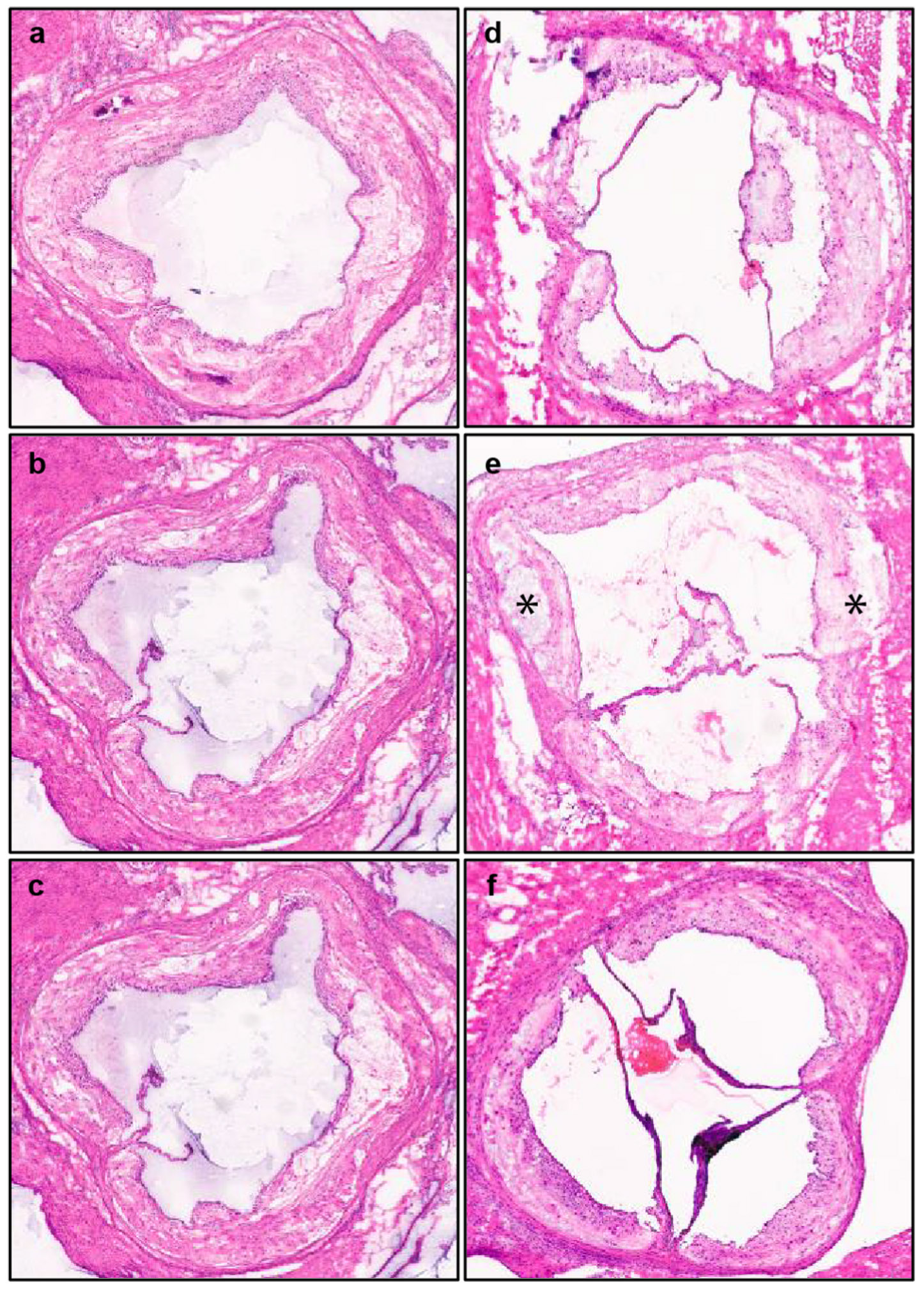
Aortic root sections **(a–c)** from a single mouse and **d-f** from three individual mice. Technical replicates show similar morphological characteristics with H&E staining. In contrast, aortic plaques in mouse **e** depicts more necrotic cores (*) than aortic root sections from WD-fed mouse **d** and **f**.

Frozen heart tissue embedded in fish gelatin is serially sectioned at 10 μm using a cryostat set at −18 to −20 °C. We use the initial appearance of the three leaflets as a landmark to obtain consistent sections of the aortic root. Tissue sections mounted on indium tin oxide (ITO) or Bruker conducting Intellislide or charged microscopic slides are stored at −80 °C until analysis using MALDI or DESI, respectively [78]. To prevent condensation-related damage to tissue sections on glass slides when removing them from the freezer for imaging, it is recommended to keep the slides on dry ice and place them on a preheated metal plate set at 37 °C for 1 min, uncovered. Warming the slides under mild vacuum in a desiccator is also recommended to avoid condensation on the slide. To minimize batch effects, aortic root sections from different mice should be mounted on the same slide for comparison. If possible, both technical and biological replicates should be considered for imaging studies. As illustrated in Fig. 6, three aortic root sections taken from an individual mouse fed a WD and spaced 50 μm apart exhibited consistent morphological characteristics when stained with H&E. In contrast, the aortic root sections obtained from different mice on the same WD diet for the same time displayed notable heterogeneity, especially section from mouse e (Fig. 6 e) containing a much higher number of necrotic cores.

Variability in aortic root sectioning, such as observing only two lobes instead of three, can introduce spatial intensity discrepancies in MSI data, potentially leading to misleading interpretations. To correct for this discrepancy, the spectral intensities are scaled by total ion current (TIC) to minimize the signal and sample thickness variations. Median and root mean square intensity normalization helps to reduce extreme fluctuations to enhance consistency among the samples. Internal standard based normalization with reference compounds is now considered the optimum way to correct for section inconsistencies [79]. For example, normalization to an internal standard in a region of interest (ROI) in the brain resulted in better accuracy compared to TIC, median, and root mean square (RMS) normalizations [80].

### 4.4. Spatial resolution, sensitivity, and quantification issues

Spatial resolution and quantification challenges are significant hurdles in the fields of metabolomics, proteomics, and lipidomics. Furthermore, variable ionization efficiency, matrix deposition in MALDI imaging (number of paths, flow rate, nozzle temperature), or sampling depth in DESI-MS can lead to inconsistencies in signal intensity, affecting data reliability. The study of small molecules frequently suffers from inadequate spatial resolution, making it challenging to characterize the chemical makeup of biological tissues because of the averaging effects of greater sample numbers, masking intricate metabolic changes. This limitation is particularly pronounced when subsampling techniques fail to capture heterogeneity in biological samples, leading to potential misinterpretations of metabolic pathways [81]. However, efforts have been made towards enhancing MSI spatial resolution from microscale to nanoscale in tissues through improvements in both instrument optics and ultra-dry spraying methods to minimize diffusion of small molecule analytes [82]. After acquiring resolution up to 1 μm or less (nano scale), accurate molecular quantification remains a significant challenge in spatial omics studies [83].

Spatial resolution and sensitivity are two traits that limit the detection of low abundant entities (lipids, peptides, glycans) in heterogeneous tissues. Spatial biologists need to pay attention to the microscopic mode in MSI to attain the nanoscale resolution to increase the coverage of low-quantity metabolites and lipids. The lipid-rich microenvironment of atherosclerotic plaques produces strong ion-suppression and matrix effects in MSI that can mask or reduce the signal from small, soluble metabolites [84]. Thus, best practice is to combine MSI with LC-MS, targeted MSI or DESI/MRM to separate the lipid background from soluble metabolite signals [85]. However, even with increased resolution, sensitivity remains a constraint for MS instruments in spatial biology. Laser post-ionization secondary neutral mass spectrometry (Laser-SNMS) increases the ionization efficiency of TOF-SIMS with high spatial resolution and sensitivity [86]. The evolution of ion guns from monatomic to small cluster ion guns has significantly enhanced intact molecular ion yields in SIMS imaging, enabling the analysis of molecular weights exceeding 3000 Da [87]. To achieve higher resolution and optimal sensitivity in MSI, it is essential to consider acquisition speed. The increased speed allows more samples to be imaged per instrument, saving time and directly reducing costs. Therefore, improving the imaging rate or speed is of the utmost importance. TOF-SIMS currently offers the fastest imaging speed, reaching up to 50,000 pixels per second. In contrast, MALDI-MSI typically operates at speeds below 40 pixels per second, while other laser-based MSI techniques have sampling rates below 20 pixels per second. DESI-MSI has an even lower acquisition speed [22]. Matrix-assisted laser desorption/ionization Fourier transform ion cyclotron resonance mass spectrometry (MALDI-FT-ICR) is best for ultra-high-resolution mass analysis but sacrifices speed, which is lower than DESI-MS and MALDI-MSI but offers direct surface analysis without matrix application [88]. With the growing demand for analyzing complex homogeneous and heterogenous biological tissues, efforts have been made to integrate MSI technology with high-resolution mass analyzers (FT-ICR and Orbitrap) with mass resolution greater than 100,000 and mass accuracy ≥1 ppm [48,89]. TOF-MSI is a commonly used analyzer for routine high-speed MSI analysis. However, there are some intrinsic constraints to mass resolution and accuracy. Recently, several ion-mobility-MSI (IM-MSI) experiments have been reported where MALDI was coupled with trapped ion mobility time of flight (TIMS-TOF-MSI) to increase the specificity of MS. Separating isobars in the mobility dimension and boosting the confidence of peak assignments are made possible by the combination of ion mobility separation capabilities with MSI [90,91]. Tandem MS/MS imaging was also developed to enhance the specific identification of metabolite which provide more specific fragment information. The identification of unknown metabolites and their isomers can be further refined by utilizing comprehensive database searches with an in-depth review of relevant literature. Metabolite databases and public libraries provide spectral reference data, structural information, and fragmentation patterns, which serve as a foundation for identification and characterization [25]. After entity identification, the accurate and precise quantification is the unavoidable challenge in the MSI study in intricate biological tissues.

The study of small metabolites often suffers from limited spatial resolution, making it difficult to characterize them in biological tissues due to the averaging effects of high abundance metabolites, which obscure detailed metabolic variations. This limitation is particularly pronounced when subsampling techniques fail to capture the heterogeneity in biological samples, leading to potential misinterpretation [81]. Similarly, in a proteomics workflow, the vast diversity and complexity of proteomic samples and the quantification of low-abundance proteins, which may be lost amid higher-abundance species during analysis present challenges [92]. The complex nature of lipid species, which can differ substantially in their chemical structure and concentration, makes quantification problems in lipidomics even more difficult. This means that distinct analytical techniques may be needed to cover the lipidome.

The normalization of ion signals to the TIC, RMS, and average intensity are widely used approaches in MSI experiments. TIC is the most common normalization strategy which is the sum of all signals present in each spectrum [93]. For more heterogenous tissues such as atherosclerotic plaques in the aorta, TIC normalization might be a difficult because it contains a variety of regions or cell types that express a diverse range of proteins, and lipids leading to distinct ion distributions. It can sometimes include matrix and background peaks, which can interfere with normalization and ultimately impact reproducibility and quantification accuracy. To address this issue, it is a common practice to calculate the TIC using only biologically relevant peaks derived from the sample of interest and normalize based on this biological TIC [94]. A recent study on MSI profiling of atherosclerotic plaques defined the ROI which was normalized against TIC [95]. Tu and Muddiman suggested that local median normalization enhances repeatability and quantitative confidence in both homogeneous and heterogeneous samples [94]. However, quantitative MSI analysis is still evolving.

## 5. Data processing and analysis

### 5.1. Spectral data processing

The preprocessing of acquired MSI spectra to improve the signal-to-noise ratio is crucial for the accuracy of subsequent analyses [96]. The preprocessing workflow, adapted to the specific experiment and objectives, generally involves steps such as baseline correction, noise reduction, spectral alignment, mass calibration, normalization, peak picking and selection, binning, and matrix-peak removal [97]. Following preprocessing, the annotation or identification of metabolites, lipids, or proteins can be performed [96,98]. Identification remains a challenging task due to the inability of MSI to generate high-quality MS/MS spectra in an untargeted manner, limiting the structural information available. Annotation based on MS1 data can be accomplished through the extensive use of various mass spectral databases [99,100]. Additionally, machine learning approaches specifically designed for MSI annotation are increasingly available [101]. The advent of ion mobility techniques, such as TIMS-TOF-IMS, has enabled the separation of isomeric lipids, with the METLIN CCS database providing essential support for their identification. This resource, which compiles information on over 900,000 molecular standards, provides valuable data for machine and deep learning methods, enabling more accurate lipid identifications in both on-tissue and off-tissue samples [102].

A wide range of open-source software is available for the processing of spectral imaging data, including several desktop applications such as mzmine [91], LipostarMSI [103], msIQuant [80], and LipidXplorer [104], as well as web applications like METASPACE [100] and OpenMSI [105]. Additionally, packages within the R/Python ecosystem, including Cardinal [106], rMSI [97], SpatialData [107], and Napari [108,109], offer extensive set of tools for spectral data analysis (Table 2). Commercial and vendor-specific software, such as MSiReader, SCiLS, Fleximaging, and HDi, are also widely used. Despite the availability of these tools, the computational demands associated with processing high-resolution MSI datasets present a significant barrier for laboratories with limited computing resources [5].

### 5.2. Combining multi-modal MSI with H&E morphology and cross-validation with LC-MS/MS

**Fig. 7.**
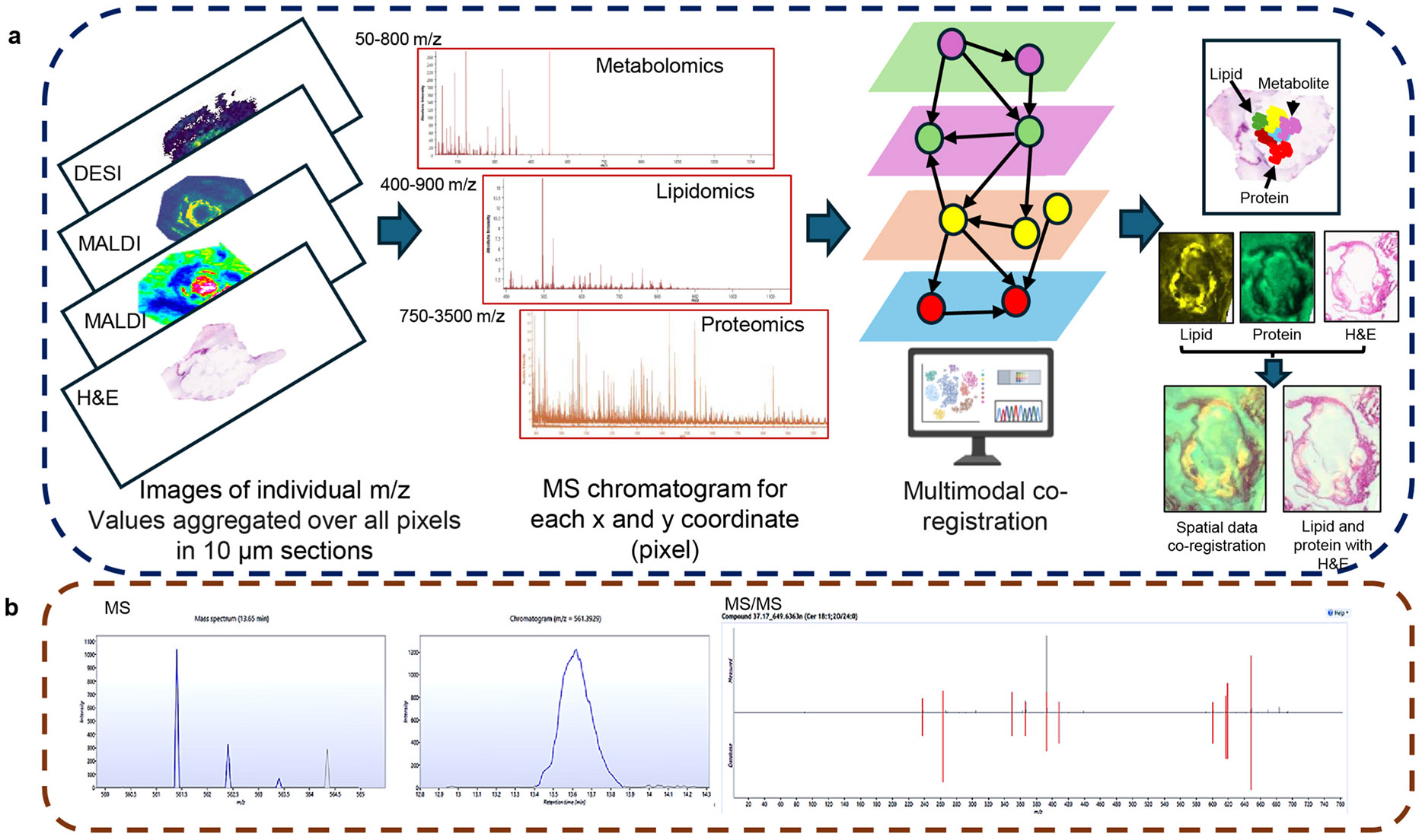
Spatial multi-omics data integration workflow **a.** and cross validation of features using LC-MS-MS showing Cer d18:1; O2/24:0 at MS1 and MS/MS fragments, **b**. Cross validation of MSI with LC-MS-MS.

A rapidly evolving area of spatial omics technologies seeks to analyze formalin-fixed, paraffin-embedded, fresh-frozen, and living tissues, with improved resolution, deeper coverage, multiplexity, and adaptability. The emerging multi-modal technologies and protocols enable the acquisition of multi-modal spectra within the same tissue section [110], unveiling a comprehensive molecular landscape that encompasses metabolites, lipids, and proteins, thereby facilitating the discovery of novel biological insights. This concept is illustrated in Fig. 7a, which demonstrates the multimodal co-registration of lipid and protein data on an H&E-stained slide. A high throughput targeted in situ sequencing method, called multi-omics in situ pairwise sequencing, simultaneously detects multiplexed DNA, RNA, proteins, and metabolites at subcellular resolution, offering extensive data for researching cellular processes and disease mechanisms [111]. Spatial omics protocols combining histology, MSI, and spatial transcriptomics are being developed to enhance the understanding of biological processes [112], alongside deep learning solutions that utilize H&E morphology and MSI data [113]. Cross-validation is essential to ensure the accuracy, reliability and biological relevance of such omics solutions (Fig. 7b).

For example, cross-validation using LC-MS/MS is essential to confirm molecular identities and ensure data reliability due to the limited MS/MS capability in MSI, matrix effects, ion suppression, and limited dynamic range. The high sensitivity and specificity of LC-MS/MS could offer absolute quantification and complements MSI by confirming the identity and abundance of metabolites, lipids, and proteins detected in tissue regions of interest. For example, LC-MS/MS validated the distribution of heterogeneous cholesterol esters in tissue extracts of atherosclerotic plaques using MSI [114]. This will also help to resolve the complexity in annotation of isomeric separation of lipids and metabolites. Such cross-validation also facilitates data integration into systems biology models that attempt to elucidate how plaque composition, structure and mechanical properties are influenced by lipid-protein interactions [115].

### 5.3. Data analysis in multi-omics studies

Analysis of metabolomics and lipidomics data often involves well established statistical and machine learning procedures, including principal component analysis (PCA), non-negative matrix factorization (NMF) and hierarchical cluster analysis (HCA) for unsupervised tasks of dimensionality reduction, feature extraction and clustering, respectively [116,117], together with orthogonal projections to latent structures discriminant analysis (OPLS-DA) and logistic regression for supervised tasks such as classification of samples based on experimental conditions [118-120]. These approaches have been extensively examined and frequently perform well, assuming the data have been appropriately transformed and normalized [121,122] and that sufficient attention is paid to phenomena such as overfitting [123]. However, application of these methods to imaging data requires careful consideration of correlation and dependence present in measurements obtained from neighboring pixels (or voxels, spots, cells, etc.). Failure to account for this dependence can lead to false positive errors due to treatment of measurement at neighboring pixels as independent, or false negative errors when excessive variance can be controlled using the spatial information. Several methods, such as spatial PCA [124] and spatial NMF [125], have introduced improvements to the aforementioned approaches, taking spatial aspects of imaging data into account. While classical techniques have many advantages, including fast execution and interpretability [126], their linear underpinnings can hit limitations when presented with complex data combining multiple modalities in a spatial setting. Deep learning and networking approaches have been successfully applied to various tasks such as multimodal data integration and inference of cell-cell communication, predominantly in the context of (spatial) single-cell transcriptomics [127]. These approaches can often be directly applied to mass spectrometry imaging data [128-132], although development of extensions that incorporate unique properties of metabolites and lipids is strongly encouraged [133,134]. We expand on these thoughts below.

#### 5.3.1. Data integration

The effectiveness of integration methods depends on the quality of the input data. Therefore, it is essential to address technical batch effects in the data, such as pixel-to-pixel, section-to-section, and slide-to-slide variations, that can introduce noise and mask biological variability across cells, tissues, and organisms [135,136]. Before proceeding with integration, it is recommended to explore the data from each individual omics layer separately, possibly using one or more spatially informed methods [124,125,137]. This is important since various technical issues-often arising from scale discrepancies-can impact the integration process. These issues include differences in the number of features, levels of noise, and measured dynamic ranges across modalities. Such challenges may lead to biased results that favor a particular modality or complicate the interpretation of the findings.

**Fig. 8.**
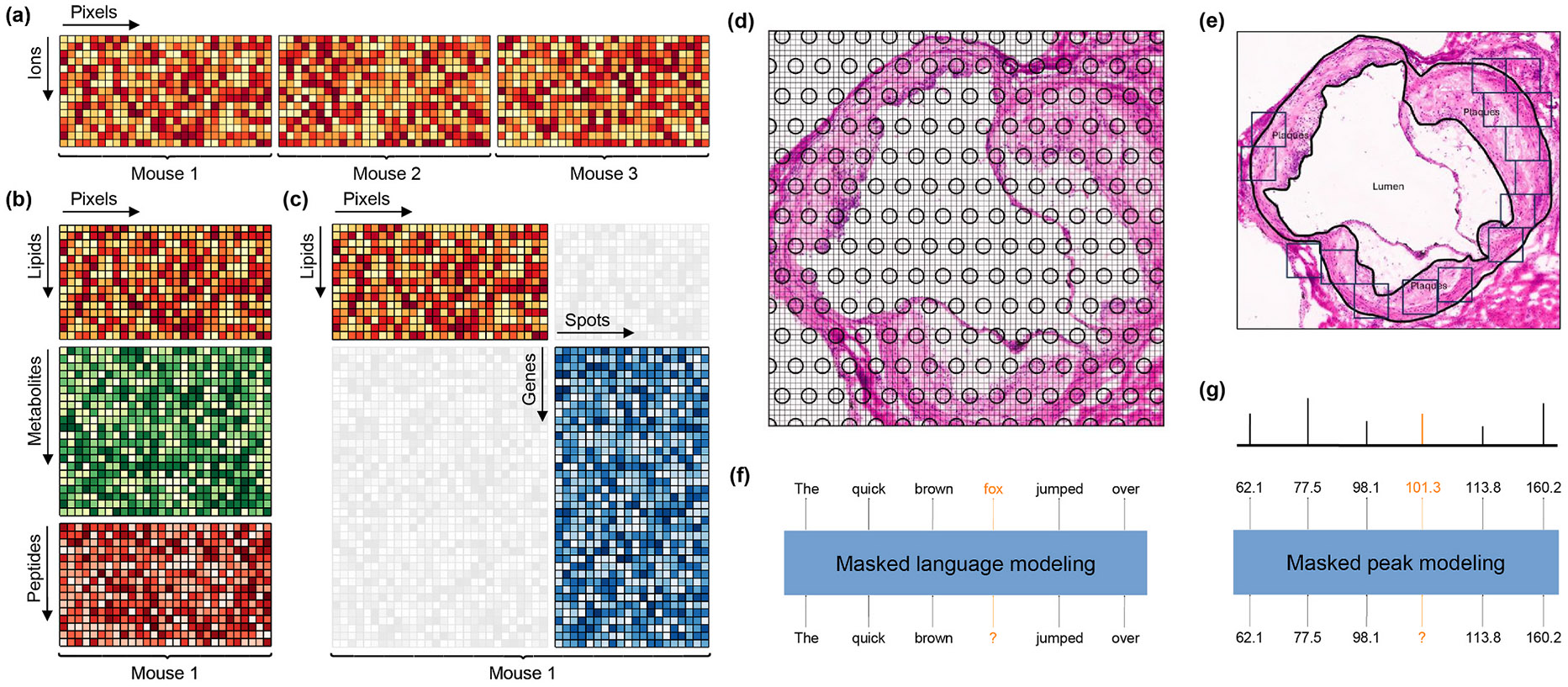
Examples of data analysis strategies in multi-omics studies. **(a)** Horizontal integration across three biological replicates: columns represent pixels, which are unwound from their spatial 2D arrangement into 1D line, rows represent ions, with color indicating the intensity of each ion at a specific pixel. **(b)** Vertical integration across three modalities measured at the same resolution. **(c)** Diagonal integration across mismatched modalities: faint regions correspond to data that were not measured (gene expression at pixels-level resolution and ion intensities at spot level resolution). **(d)** Hematoxylin and eosin (H&E) staining of a mouse aortic root, with 10x Genomics Visium 55 μm diameter circular spots and MALDI-MSI 20 μm wide lattice overlaid. **(e)** H&E staining of mouse aortic root, showing selected 100 μm wide regions of interest for image-based spatial transcriptomics. **(f, g)** Masked modeling in large language models **(f)** and metabolic models **(g)**.

Several distinct data integration tasks exist, each characterized by unique approaches and challenges. Horizontal integration combines measurements of one set of features across different samples or batches (Fig. 8a). In the context of imaging data, horizontal integration can be challenging due to the difficulty of comparing spatial distributions of features across samples, necessitating the development of alignment methods [138-140]. Vertical integration (Fig. 8b), on the other hand, focuses on merging features across multiple modalities (e.g., transcriptomics and lipidomics). Although several MSI technologies are currently available that assay multiple modalities on the same section [53,112], most typically probe only one modality per (adjacent) section. As a result, vertical integration in this setting is more complex and resembles diagonal integration, wherein both the features and measured entities (pixels, spots, cells, etc.) differ across datasets [141] (Fig. 8c).

Integration of spatial transcriptomics with MSI data also requires careful consideration of resolution discrepancies. For instance, the 10x Genomics Visium platform captures transcripts within circular spots that have a diameter of 55 μm and are spaced 100 μm apart, whereas MALDI MSI collects data across a finer lattice with 20 μm pixels (Fig. 8d). When studying atherosclerosis in a mouse model, the small size of the mouse aortic root (~1 mm in diameter) may preclude the use of the Visium platform, as it would result in a limited number of spots covering the aorta (Fig. 8d). Although the 10x Genomics Visium HD platform [142] offers significantly higher resolution with 2 μm pixels, it may be cost-inefficient, as the lumen does not need to be assayed. Certain imaging-based protocols, which provide cellular or sub-cellular resolution, allow for the preselection of regions of interest for analysis, which can be particularly advantageous given the circular shape of the aorta (Fig. 8e). Regardless of the protocol chosen [143], the resolution of spatial transcriptomics data may be considerably higher or lower than that of MSI data. This issue can be addressed by aggregating higher-resolution data to align it with lower-resolution data [144,145]. The processing and preparation of spatial transcriptomics data prior to integration typically involve several critical steps, which have been extensively discussed elsewhere [146].

Several methods initially developed for the vertical and diagonal integration of non-spatial data, such as weighted-nearest neighbor [147] and matrix factorization techniques [148,149], have been adopted for integration tasks involving spatial transcriptomics and mass spectrometry imaging data [144,150]. Nevertheless, emerging approaches are increasingly focused on directly incorporating spatial information. These methods leverage a wide range of machine learning techniques, including optimal transport [151] and graph neural networks [152]. It is expected that various manifold learning approaches, such as autoencoders [153], will also be employed to address the challenges associated with spatial multi-modal data.

Finally, we mention several ongoing challenges in data integration and potential avenues for future improvement. In addition to the general issues with integration approaches discussed in previous works [154-157], we emphasize that, to date, only a limited number of studies-primarily focused on cancer-have attempted to integrate MSI with spatial transcriptomics data. Consequently, it is crucial for researchers to evaluate critically the biological coherence of results derived from various integration approaches and remain vigilant to emerging technologies that may overcome the technical limitations of the current ones. Benchmarking efforts [158,159] are encouraged to incorporate lipidomics and metabolomics data from MSI and offer guidance to researchers in selecting appropriate technologies and integration strategies, although it is important to recognize that obtaining ground truth data-essential for both benchmarking and deep learning approaches-is challenging and costly [134,160]. From a practical perspective, it is encouraging to witness the development of several core spatial data analysis frameworks that facilitate the handling and investigation of complex multi-omics data sets [107,161,162].

#### 5.3.2. Downstream analyses

A range of analyses can be performed on individual data sets or on integrated multi-omics data, depending on the specific research questions being addressed. Spatially variable genes, lipids, or metabolites can be examined, and spatially aware dimension reduction and clustering algorithms employed to represent visually the data and delineate distinct spatial domains based on cellular presence or activity. Trajectory and RNA velocity analyses provide valuable insights into cell-state transitions and ultimate cell fate. Algorithms designed to leverage spatial transcriptomics data for such analyses are available [136,146]. Some of these algorithms are modality-agnostic and can be applied to metabolomics or lipidomics data. However, in many instances, the full potential of additional modalities is best realized through specialized techniques, such as in enrichment analyses [101]. In the context of atherosclerosis research, the inference of cell-cell communication and interaction is of interest. Several existing approaches utilize spatial transcriptomics data for this purpose [136,146]. There is significant potential to enhance these approaches by incorporating information on oxylipins and other cell-signaling lipids or metabolites, as well as glycomic data, due to the impact of glycosylation on ligand-receptor affinity [163]. However, current MSI technology is limited in its ability to capture oxylipins [164].

As previously noted, benchmarking efforts have yet to keep pace with the availability of new modalities, and obtaining ground truth data remains challenging [134,146]. However, benchmarking is essential to ensure the accuracy and reliability of both novel approaches and those that have primarily been tested on, for instance, transcriptomics data. Finally, it is important to recognize that highly non-linear deep learning methods may suffer from limited interpretability. This may present an opportunity for foundation models, which are discussed in the following section.

#### 5.3.3. Foundation models

Foundation models are deep learning models trained on vast amounts of (generally unlabeled) data that can be employed to solve various tasks, either directly, or after some adjustment which requires a considerably smaller amount of task-specific data [165], such as cell atlases which catalog cell-specific transcriptomics [166]. The performance of early models [167] on specific downstream tasks, such as cell-type annotation, did not exceed already established methods [168], but more recent models trained on even bigger data sets show promise [169]. While many current models rely exclusively on transcriptomics data, some go beyond this by utilizing already existing protein foundation models [170] or including information about chromatin accessibility in their training set [171].

Foundation models are often trained using some variation of the masking technique, where a model attempts to predict withheld information, e.g., a missing word in a sentence (Fig. 8f). In this vein, models can be trained by masking parts of DNA or amino acid sequence [172], or a certain percentage of the gene expression data of a cell [173]. Ideally, trained models can be specialized to solve particular tasks, such as peptide property prediction [174], cell similarity search [175], or prediction of effects of intervention on the transcriptional state of a cell [176]. In the realm of mass spectrometry, models can be trained by masking peaks in a spectrum (Fig. 8g). However, current attempts are focused on MS/MS spectra [177,178], which are difficult to obtain with current MSI technology. Although foundation models are rapidly evolving [179], with the prospect of incorporating spatial [180] and perturbation [176] data, their validity and usefulness remain to be seen. The development of proper benchmarking techniques will be key to delivery of their promised goals in biomedicine.

### 5.4. Software and reproducibility

Software for processing and analyzing omics data is available in several forms, including desktop applications with graphical user interfaces (GUIs) that can be installed on local computers, web-based applications accessible via a web browser, and packages for statistical or programming languages such as R or Python. Each option presents its own set of advantages and disadvantages. Desktop applications do not require data to be transferred or shared, which can be particularly important during the early stages of research. However, processing large volumes of imaging data may demand computational resources that surpass the capabilities of local machines. In contrast, web applications are hosted on powerful servers capable of handling large datasets but necessitate the transport and sharing of data with external parties. R and Python packages offer particular advantages, as they can be run on both local systems and more powerful high-performance computing (HPC) and cloud infrastructure. Additionally, they are parts of extensive ecosystems, allowing researchers to integrate and utilize a wide array of supplementary packages, which significantly enhances their functionality beyond the capabilities of desktop or web applications. The primary disadvantage of R/Python packages is that they are less userfriendly, requiring researchers to invest time in learning the tools and workflows necessary for their effective use. This challenge can be mitigated through collaboration with bioinformaticians.

#### 5.4.1. Computational reproducibility

Computational reproducibility ensures that consistent results are obtained when identical computational methods are applied to the same data [181-183]. The choice of software can significantly impact computational reproducibility. While desktop and web-based applications provide convenience, they can conceal complex processing pipelines behind their graphical user interfaces (GUIs). Over time, modifications to algorithms, options, and parameters may occur behind the scenes, while the GUI remains unchanged, potentially undermining computational reproducibility. Although documenting the versions of software used is crucial, such information is often unavailable for many web applications. Furthermore, older versions of commercial software may become inaccessible in the future, underscoring the importance of utilizing open-source software to ensure long-term computational reproducibility.

Compared to desktop or web applications, the R and Python ecosystems offer several advantages. The most notable of these is their ability to define and specify complex data processing and analysis pipelines through text-based scripts, which can be easily shared among researchers. Additionally, most packages within these ecosystems are open-source and distributed under permissive licenses. To ensure reproducibility when using this approach, it is essential to report the versions of the packages used in the analysis, while a more robust strategy would be to encapsulate the R/Python executable along with all necessary packages for pipeline execution within a container image [184]. Containerization facilitates the execution of data analysis pipelines by other researchers, provided that the data are accessible [185].

For computational reproducibility, it is crucial that data be easily findable and accessible [186]. Publicly available data repositories, with METASPACE [100] being the primary platform for MSI data, enable this accessibility. However, the broader scientific impact also relies on the data being interoperable and reusable, as emphasized by the FAIR principles [186]. Achieving interoperability and reusability hinges on the adoption of open data formats, such as imzML [187], mzTab-M [188], and others [96], which not only ensure the seamless exchange and integration of data across various platforms and analytical tools, but also promote long-term sustainability and foster collaborative research efforts, thereby reducing the risk of vendor lock-in and encouraging a more open, adaptable scientific environment.

#### 5.4.2. Replicability of experiments

A replicable experiment will yield consistent results when independently repeated using the same methodology [189]. As the repeated experiment typically involves animals or human subjects not included in the original study, it is essential to measure variability across biological replicates and appropriately account for it in the data analysis. Imaging and single-cell protocols generate a large number of measurements for each probed feature-the intensity of a specific ion is assessed across thousands of pixels, while the expression of a particular gene is quantified across thousands of cells. Comparing two or more conditions using established statistical methods, such as the *t*-test or ANOVA, may result in misleadingly low p-values if measurements from individual pixels or cells are treated as independent [190]. To ensure replicability, it is crucial to apply analysis methods that place due emphasis on measurements from distinct biological replicates (cf. Fig. 6d-f). A straightforward approach involves aggregating measurements within a defined region of interest (e.g., a plaque) for each replicate, followed by a comparison of the resulting values across conditions, similar to the pseudobulk method used in single-cell transcriptomics analysis. More advanced methods that incorporate spatial or cell-type information are needed, yet remain relatively scarce [191].

The integration of MSI with emerging spatial multi-omics platforms represents a promising direction for atherosclerosis research. The development of tools that combine MSI data with spatial transcriptomics and proteomics will provide unprecedented insights into the cellular and molecular mechanisms underlying plaque development and rupture. As these technologies continue to evolve, the development of standardized workflows and accessible data analysis solutions will be essential for realizing the full potential of spatial omics approaches in cardiovascular disease research.

## 6. Future guidelines, opportunities, and perspectives

### 6.1. Advances in instrumentation and AI-driven analysis for data integration

When integrated with advanced web interfaces for automated tissue morphology segmentation and cellular or subcellular analysis, MSI enables the simultaneous per-cell quantification of multiple markers. Recent advances in MALDI-MSI combined with immunofluorescence analysis on single tissue sections have enabled the spatial correlation of lipid alterations with brain histological regions, revealing amyloid plaque-associated changes in glycerophospholipids and sphingolipids [192]. MSI is among the many scientific domains where artificial intelligence (AI) is revolutionizing data analysis. The capabilities and efficiency of this approach have been significantly increased by developments in MSI instrumentation combined with AI-driven data integration multi-omics techniques. By introducing machine learning and deep algorithms into the MSI workflow, researchers have started to develop new strategies to evaluate and comprehend the massive volumes of data produced by this technology. Earlier, approaches relied on manual peak selection and prerequisite algorithms have produced biased and inconsistent results [129]. AI-driven practices can play a pivotal role in providing for feature extraction and unsupervised analysis from MSI data to uncover hidden structures [134].

A recent study used an AI model involving machine learning algorithms for single-section multimodal imaging in DESI-MSI and correlating metabolic features with phenotypic characters [193]. AI-driven analysis improves not only molecular feature identification but also integrates them into unified datasets allowing comparative studies across different tissue samples and conditions. AI methods systematically reveal the interconnection between spatial registration and metabolic signatures in different tissue classifications. Furthermore, MSI and AI-driven integrated tools make these sophisticated technologies more readily available for those who might not have a computational background. Another recent effort has been made for an open-source end-to-end platform for MSI with AI-driven data analysis software MassVision provide end-to-end MSI analysis solutions, allowing for straightforward data visualization, curation, and integration. MassVision was applied usefully across a variety of applications using in-house and publicly available data from different MSI modalities [194].

### 6.2. Spatial mapping of oxylipins is challenging but represents an opportunity in atherosclerotic plaques

Emerging evidence also suggests the engagement of oxylipins in inflammation is a core process in atherosclerosis. Atherosclerotic lesions exhibit enhanced oxylipin production, which may drive or restrain further inflammatory cytokine secretion and recruitment of immune cells to the arterial wall [195]. Thus, the role of oxylipins in atherosclerosis may reflect not only their biochemical properties but also their interactions with various cellular signaling networks, contributing to local inflammation within arterial tissues and systemic metabolic dysregulation. Innovative lipidomic profiling techniques have been invaluable in identifying distinct oxylipin signatures associated with atherosclerosis and related cardiovascular conditions, advancing our understanding of their role as biomarkers for disease states [196]. Recently reported spatial MSI methods using a DESI-multiple reaction monitoring (MRM) workflow allowed mapping of low abundance oxylipins in pulmonary tissue [164]. Isomeric separation of oxylipins presents a significant challenge in spatial MSI. TIMS-TOF plays a critical role in effective separation of isomeric oxylipins and preventing overlap in mass spectra, a process that remains challenging in MSI [197]. Their precise identification is a valuable direction for future research. Oxylipins are difficult to detect using spatial technologies. However, one study using MSI investigated the localization and formation of isomeric PGE2 and PGD2 at the implantation site in mice, coincident with immunostaining of COX-2 expression [198]. However, no study to date has evaluated the oxylipin profile of aortic plaque tissue using MSI.

### 6.3. Cellular and sub-cellular analysis

The future of cellular and sub-cellular imaging using MSI holds immense potential for advancing spatial omics, offering molecular insights at single cell (Sc) resolution. Sc analysis is generally performed at spatial resolution of ≤5 μm. High resolution MALDI and nanoDESI coupled with advanced ion mobility have achieve spatial resolution to allow cellular imaging up to Sc resolution [199,200]. This makes MSI the more powerful tool. High-resolution spatial mapping of intercellular and cell-to-cell lipidome heterogeneity, pharmacologically-induced Sc lipidome remodeling, and region-specific lipid diversity within tissue have been made possible by Sc-MSI technology [201]. With the development of flexible, effective, and precise spatial segmentation workflows such as Sag:MSI-a graph based segmentation approach that integrates spatial-aware graph construction of MSI data with a classical graph convolution network (GCN) module in a deep neural network [202]. This strategy is expected to be applicable to other omics fields, including single-cell metabolomics and spatial transcriptomics, enabling multimodal omics integration.

Integrating AI-driven image analysis and deep proteomic characterization will provide key insights within tissues for the spatial organization of the proteome [203]. This convergence of technologies promises to revolutionize our understanding of tissue biology, disease processes and drug responses at the cellular and sub-cellular levels.

## 7. Conclusions

This review provides an overview of how spatial MSI can enhance our understanding of atherosclerosis in conjunction with existing omics approaches. MSI has demonstrated remarkable potential to identify various analyte classes and distinct biomarkers in complex heterogenous atherosclerotic plaques at different stages of disease evolution.

This review also highlights the challenges for future applications of multimodal MSI, particularly in relation to tissue preparation and data integration. As MSI technologies advance, the acquisition of acquiring fast MS/MS data in spatial platforms holds substantial promise for our understanding of disease pathogenesis and identification of drug targets and predictive biomarkers at a personalized level to augment our ability to mitigate the burden of cardiovascular disease. Although many of the cited papers illustrate the promise of these technologies and the prospects of their integration, the field is still at an early stage. Thus, most studies are small, few integrate disparate streams of data-either technologically or across the translational divide - and none include longitudinal data sets relating measurements to clinical outcomes at scale. However, progress is rapid and AI approaches to data analysis and integration have served as catalysts to developments in the field.

## Figures and Tables

**Table 1:** Comparison of MSI methods and their features.

MSI technique	Sample preparation	Routine spatial resolution	Molecular mapping	Routine acquisition speed	Cost and accessibility (Source + MS)	Advantages
MALDI-MSI (Matrix-Assisted Laser Desorption Ionization)	Matrix deposition (α-CHCA, DHB etc.)	~1–20 μm	Lipids, metabolites, proteins, peptides, drugs	Fastest (2–100 pixels/sec)	~$400–800K Moderate to high cost; widely available in core facilities; requires matrix consumables	High sensitivity, wide molecular coverage, good for complex tissues
DESI-MSI (Desorption Electrospray Ionization)	Minimal, no matrix required	~5-100 μm	Lipids, metabolites, small molecules, peptides, drugs	Fast (10–50 pixels/sec)	~$300–600K Moderate cost; growing availability; minimal consumables; simpler operation	Ambient ionization, no complex sample preparation
SIMS-MSI (Secondary Ion Mass Spectrometry)	Rigid and conductive coating may be required	~50 nm-1 μm	Small molecules, lipids, elements, fragments of larger biomolecules	Fast (1K pixels/sec)	~$1.5–2.5 M High cost; limited availability; specialized facilities only	Ultra-high spatial resolution and sensitivity
LAESI-MSI (Laser Ablation Electrospray Ionization)	Requires water-rich samples	~100–200 μm	Metabolites, lipids, saccharides	Moderate (1–5 pixels/sec)	~$200–400K Moderate cost; not commercially available; custom setups only	Direct analysis of hydrated/biological samples, minimal sample preparation
Nano-DESI-MSI	Minimal, no matrix required	~7–10 μm	Lipids, metabolites, drugs	Moderate (5–15 pixels/sec)	~$300–600K Moderate cost; not commercially available; custom setups only	High spatial resolution, minimal sample preparation
AP-MALDI MSI (Atmospheric Pressure MALDI)	Matrix deposition (α-CHCA, DHB etc.)	~10–50 μm	Lipids, metabolites, peptides	Moderate (1–5 pixels/sec)	~$300–600K Moderate cost; requires matrix consumables	Highly portable, easily switched with other sources, softer conditions, compatible with fragile biomolecules

**Table 2:** Mass spectrometry imaging free and commercial software for data analysis.

Name of software	Year of release	Released by	Free/ Commercial	Advantages or key features
QMSI	–	Open source	Free	Data visualization, ROI, statistical analysis
Mzmine	2010	Open source	Free	Data visualization, ROI, peak annotation, pathway and network analysis
PREMIERBiosoft	2012	PREMIER Biosoft	Commercial	Data visualization
MSi Reader	2013	Open source	Free	Data visualization, ROI, statistical analysis
OmniSpect	2013	Open source	Free	Data visualization and analysis
Cardinal (R package)	2014	Open source	Free	Peak picking, supports multi-omics, visualization, statistical analysis
Datacube Explorer	2014	Open source	Free	Data visualization
HDI Imaging	2015	Waters	Commercial	Data visualization and analysis
MetaboScape	2016	Bruker	Commercial	Peak annotation, data visualization, support multi-omics, annotation with databases
MSIquant	2016	Open source	Free	Data visualization, ROI, statistical analysis
METASPACE	2016	EMBL (European Molecular Biology Laboratory)	Free	Peak picking, peak annotation, data visualization
SpectralAnalysis	2016	Open source	Free	Data visualization, ROI, feature annotation, statistical analysis
SciLS Lab	2017	Bruker	Commercial	Basic analysis, peak picking, data visualization, statistical analysis
MALDIquant (R package)	2017	Open source	Free	Data visualization and statistical analysis
LipidSpace	2017	Open source	Free	Data visualization, ROI, feature annotation, support multiomics, statistical analysis
Mass Imager	2018	Minzu University of China	Commercial	Data visualization
eMSI	2019	Open source	Free	Data visualization, statistical analysis
Metaboscape (with ccs)	2022	Bruker	Commercial	Peak picking, data visualization, support multi-omics, annotation with databases, Ion-mobility support
SpatialData	2024	Open source	Free	Python framework for processing spatial omics data
JuliaMSI	2025	Open source	Free	Computationally efficient, userfriendly environment for large-scale MSI analysis
MSI.EAGLE	2025	Open source	Free	Data visualization, statistical analysis, peak picking, data visualization, annotation with databases
Napari (with MSI-Explorer plugin)	2025	Open source	Free	Interactive viewer for multidimensional images in Python

## Data Availability

No data was used for the research described in the article.

## HIGHLIGHTS

- MSI is a powerful technique that enables spatially resolved mapping of lipids and proteins in atherosclerotic plaques.
- MSI is a tool to investigate region specific alterations in atherosclerotic plaques.
- Challenges to achieve cellular and subcellular resolution in MSI.
- Multimodal approaches will enhance insights into disease progression and plaque vulnerability.
- High-throughput annotation and machine learning-based data integration tools need further development.
